# Pesticide Use and Degradation Strategies: Food Safety, Challenges and Perspectives

**DOI:** 10.3390/foods12142709

**Published:** 2023-07-15

**Authors:** Andreja Leskovac, Sandra Petrović

**Affiliations:** Vinca Institute of Nuclear Sciences-National Institute of the Republic of Serbia, University of Belgrade, M. Petrovića Alasa 12-14, 11000 Belgrade, Serbia; andreja@vin.bg.ac.rs

**Keywords:** organophosphate pesticides, pesticide use, biotic degradation strategy, abiotic degradation strategy, food safety

## Abstract

While recognizing the gaps in pesticide regulations that impact consumer safety, public health concerns associated with pesticide contamination of foods are pointed out. The strategies and research directions proposed to prevent and/or reduce pesticide adverse effects on human health and the environment are discussed. Special attention is paid to organophosphate pesticides, as widely applied insecticides in agriculture, veterinary practices, and urban areas. Biotic and abiotic strategies for organophosphate pesticide degradation are discussed from a food safety perspective, indicating associated challenges and potential for further improvements. As food systems are endangered globally by unprecedented challenges, there is an urgent need to globally harmonize pesticide regulations and improve methodologies in the area of food safety to protect human health.

## 1. Introduction

As the world’s population grows, the industrialization of agriculture and the expansion of livestock production to meet increasing food demand create opportunities and challenges for food safety. These challenges place more responsibility on food manufacturers and processors to ensure food safety, preventing food contamination before it reaches the consumer [1].

**Figure 1:**
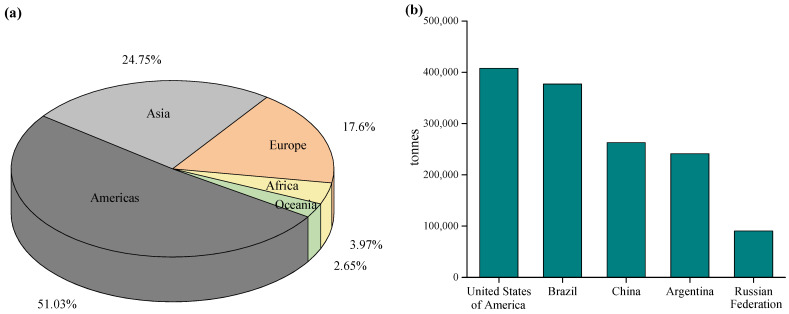
(**a**) Pesticides use by region and (**b**) the top five pesticide users in 2020.

The continuous development of agriculture intensifies the application of pesticides globally to reduce crop yield losses and increase productivity and product quality [2]. Proximately 2 million tons of pesticides are currently applied to crops worldwide each year to increase productivity and reduce losses from pests and diseases [3]. According to the Food and Agricultural Organization (FAO) of the United Nations, the United States of America was the largest user of pesticides in 2020, while the next 10 largest pesticide users in the world are Brazil, China, Argentina, the Russian Federation, Canada, France, Australia, India, and Italy [4]. In the 2022 update, FAO reported that total pesticide use in China significantly decreased, moving China to third place in pesticide usage globally [4]. However, even though a plateau has been reached in recent years, total pesticide use has increased by approximately 50% compared to the 1990s [4]. The pesticide use by region and the top five largest pesticide users in the world are shown in Figure 1.

Pesticide regulatory systems established to protect humans and the environment vary from country to country [5]. This variability implies that each country can adopt regulations to define acceptable concentrations of particular pesticides in food and feed and restrict or prohibit the usage of particular pesticides due to their unacceptable health or environmental effects. The Joint Meeting on Pesticide Residues (JMPR) is an expert body established mutually by the FAO and the World Health Organization (WHO) that is responsible for establishing toxicological endpoints, such as acceptable daily intake (ADI) and acute reference dose (ARfD), based on experimental data. Additionally, the JMPR recommends the maximum concentrations of pesticide residues (maximum residue levels, or MRLs) in food and feed to the Codex Committee on Pesticide Residues (CCPR) for consideration [6]. The recommended MRLs in food and feed that are considered safe for consumers were finally adopted by the Codex Alimentarius Commission. The MRLs, which provide a wide margin of safety based on good agricultural practice, are the most implemented standards regarding food safety [7]. However, regardless of the prevailing framework the Codex provides, the MRLs differ considerably across countries [8].

In the EU, the European Commission regulation 396/2005 directly concerns public health, establishing a system of setting and monitoring the MRLs in food and feed [9]. In the USA, the Environmental Protection Agency (EPA) is responsible for the pesticide registration, regulations, and establishment of MRLs in food and feed following the Federal Insecticide, Fungicide, and Rodenticide Act (FIFRA) and the Federal Food, Drug, and Cosmetic Act (FFDCA) [10]. The US Department of Agriculture (USDA) and the Food and Drug Administration (FDA) are responsible for measuring and collecting data on pesticide residues in fruits, vegetables, grains, meat, and dairy products nationwide and in products imported from other countries. In China, the Ministry of Agriculture and Rural Affairs (MARA) is the main pesticide regulatory body responsible for pesticide registration and management [11]. In Brazil, pesticide regulations are supervised by the Ministry of Health through the National Sanitary Surveillance Agency (ANVISA), the Brazilian Institute for the Environment and Renewable Natural Resources (Ibama), and the Ministry of Agriculture, Livestock, and Food Supplies (MAPA) [12].

Of the total amount of pesticides used worldwide, organophosphate pesticides (OPs) account for approximately 33% [13]. As effective and broad-spectrum insecticides, they are extensively used worldwide in agriculture, homes, gardens, and veterinary practices [14]. In the last decade, over 100 organophosphorus compounds have been commercially used as insecticides to control pests in agricultural food commodities [15], of which the medium- or low-toxic OPs, such as dimethoate, phoxim, chlorpyrifos, and trichlorfon, are widely used [16]. Although numerous OPs are no longer approved in most developed countries, they are still in use in many developing countries, causing long-term negative effects on human health and the environment [7]. Acute and/or chronic exposure to OPs can occur directly from occupational and non-occupational use and indirectly through the consumption of pesticide residues that can remain in food and drinking water [17]. Pesticide residues and their metabolites can contaminate soils and water, enter the food chain, and, as a final point, display toxic effects, affecting human health [16,18,19].

The increased quantity and frequency of pesticide utilization worldwide consequently increased their impact on the environment and human health. The excessive use and misuse of pesticides, especially in developing countries, can cause environmental pollution and adverse human health effects in the long run. While recognizing gaps in pesticide regulations that impact consumer safety, public health concerns related to pesticide contamination of foods and recent strategies proposed to prevent and/or reduce their adverse effects on human health and the environment are discussed. Particular attention is paid to biotic and abiotic strategies used for OPs degradation, identifying challenges and potential for future improvements. 

References for this manuscript, published by June 2023, were collected from scientific databases (PubMed, Science Direct, Scopus, Google Scholar, Taylor & Francis platform, and BioMed Central platform), using the keywords “organophosphate pesticides”, “pesticide use”, “food safety”, “pesticide regulations”, “human health”, “environmental pollution”, “pesticide detection”, “biotic degradation”, “abiotic degradation” and combinations thereof. The relevant, up-to-date peer-reviewed articles, published in English, addressing current pesticide degradation strategies and public health concerns related to pesticide exposure and food safety are included. In addition, grey literature relevant to the topic, including selected reports of government agencies and international organizations, is incorporated. 

## 2. Public Health Concerns Related to Pesticide Exposure

One of the major issues related to food safety is the lack of globally harmonized pesticide legislation and safety standards [20]. Pesticide MRLs in foods and feeds significantly differ, especially among developed and developing countries. The differences in regulations also cause trade issues since many developing countries use unauthorized pesticides or different MRLs [20]. Also, the EU MRLs are more stringent than the Codex MRLs, raising concern about whether the Codex MRL values sufficiently protect consumer health [20]. Most developed countries established their own MRL policies, and for developing countries, meeting the MRL requirements of developed countries can be particularly challenging [21].

Pesticide poisoning and mortality occur mostly in developing countries and are usually associated with insufficient occupational safety standards and regulations, inadequate application, and poor labeling of pesticides [22]. Inadequate regulatory systems also result in the import of pesticides banned in developed countries, while a lack of awareness among farmers and inadequate personal protective equipment cause poor pesticide practices. According to a report by the European Parliament (2021) [7] and Pesticide Atlas Kenya Edition (2022) [23], many pesticides no longer authorized in the European Union are still allowed to be manufactured and exported in developing countries. For example, until its ban in 2020, chlorpyrifos was the most commonly used pesticide in food production in the EU [24]. However, it is still being applied in China, India, and many other countries of the Global South [24,25]. Brazil, the largest pesticide consumer in Latin America, approved 475 new pesticides in 2019, of which about a third contain active substances that have been banned or restricted in the EU [7]. In 2019, Brazil imported 14 hazardous compounds, including chlorpyrifos, fipronil, cyanamide, and propineb [23]. Kenya, as a major importer of banned pesticides mainly from the EU and China, has registered 51 active ingredients prohibited in the EU, such as trichlorfon, atrazine, fipronil, iprodione, acetochlorines, and 1,3-dichloropropene [23]. The United States also allows the production and export of domestically banned pesticides to low- and middle-income countries where they have been linked to significant adverse health effects on the local population [26]. In addition, food containing residues of banned pesticides is frequently reimported back to the countries that allow their production and export, contributing to a global pesticide exposure risk [23]. To address the gap in the regulations of pesticides that pose risks to human health and the environment, in 2020, the European Commission drafted a legislative initiative to prohibit the production and export of hazardous chemicals banned in the EU, which is expected to come into force in 2023 [23].

There are numerous reports indicating pesticide contamination of foods. For example, an earlier study from Ghana reported that chlorpyrifos, diazinon, deltamethrin, fenvalerate, and permethrin concentrations exceeded their respective EU MRLs in some ready-to-eat vegetable samples collected from different sites along the food chain [27]. Similar results were obtained in the study of 160 samples of commonly consumed fruits and vegetables collected from all supply chain stages (distribution, storage, and handling from farm to fork) in the Kampala Metropolitan Area, Uganda. In 95.6% of the samples, multiple pesticide residues were detected, of which 91.3% were organophosphates [28]. The analysis of 1183 bovine milk samples from different locations in India demonstrated that approximately 8% contained organochlorines, organophosphates (ethion, profenofos, chlorpyrifos), synthetic pyrethroids, and phenylpyrazole residues, exceeding the MRL values. Chlorpyrifos was the most common OP detected [29]. Moreover, the residues of hexachlorocyclohexane (HCH), dichloro-diphenyl trichloroethane (DDT), and endosulfan were also found in some of the milk samples, although their usage was restricted or banned [29]. A recent study from Egypt reported that approximately 40% of the pesticide residues detected in samples of vegetables and fruits from the market exceeded the permissible MRLs. The most frequently detected pesticides were insecticides; the results obtained for lambda-cyhalothrin, fipronil, dimethoate, and omethoate in spinach, zucchini, kaki, and strawberry, respectively, indicate they may cause acute or chronic poisoning when consumed in amounts equal to 0.1 or 0.2 kg per day [30]. Another study from Egypt reported the presence of multiple pesticide residues (cypermethrin, thiamethoxam, chlorpyrifos, and lambda-cyhalothrin) in strawberry and tomato-based products available on the market. It was found that 27% of the average pesticide residues in the tested samples exceeded the maximum residue levels (MRLs) [31]. A recent study from Algeria has revealed the contamination of honey samples with OPs (methyl parathion, coumaphos, and fenitrothion), exceeding the MRL (MRL 50 ng/g) [32].

As mentioned, pesticide MRLs in food imported from outside the EU are generally higher than in foods from EU countries [33]. However, an enhanced level of pesticide residues in foods was also reported in EU countries. For example, a previous study from Poland reported an exceedingly high presence of chlorpyrifos in all of the investigated fruits and vegetable peels and also a high level of methyl parathion, especially in the peel of potatoes and pulp of zucchini [34]. Recent research from the UK has shown that out of the total 33,911 analyzed samples from imported foods (including from EU countries), 50.2% contained detectable residues, and 3.3% of the total analyzed samples were above MRLs [35]. Also, the contamination of foodstuffs, such as honey, with OP residues was reported in studies conducted in Italy, Spain, Belgium, France, Germany, Switzerland, and from outside Europe, such as South America and North America [36]. A recent study on more than 200 cereal and legume samples from Italy, Eastern Europe, and some non-European countries has reported the presence of pesticide residues in the grain samples (contamination percentage of 7%), which were below the MRLs, while no pesticide was found in the analyzed legumes. The most abundant pesticides in cereal samples were cyfluthrin, deltamethrin, phenothrin, cypermethrin, fenvalerate, chlorpyrifos, and pirimiphos-methyl [37].

The latest EFSA annual report, considering the assessment of pesticide residue levels in foods on the European market in 2021, has shown that 96.1% of the samples analyzed were below the MRL, while 3.9% exceeded this level, of which 2.5% were non-compliant [38]. The MRL exceedance and non-compliance rates were lower than those reported in 2020 (the MRL exceedance rate of 5.1% and the non-compliance rate of 3.6%). However, samples imported from third countries showed a 5-fold MRL exceedance rate (10.3%) and non-compliance rate (6.4%) compared to the EU-derived samples, which showed 2.1% MRL exceedance and 1.3% non-compliance [38]. Given the safety margins incorporated into the ADI and ARfD, the MRL exceedance does not necessarily imply a risk to human health, so case-by-case assessments are required to determine whether dietary intakes exceed the health-based limits. The EFSA report shows that no consumer intake concern was identified in the chronic health risk assessment. However, out of the total samples analyzed under the acute assessment, 1.1% exceeded the health-based guidance values (HBGVs) in 29 pesticides out of the 190 analyzed [38]. 

As expected, food products in developed countries are systematically monitored for pesticide residues to ensure compliance with national legislation and consumer safety. In contrast, the monitoring of food in developing countries is often restricted; nevertheless, this issue is also reported in developed countries, as shown in the case of the US, where the FDA inspects only 1–2% of import shipments [20]. Therefore, an increasing public health concern associated with pesticide contamination of food is completely justified and points out the necessity to globally harmonize and standardize MRLs to ensure consistent and effective food safety regulations worldwide. Establishing uniform MRLs is a fundamental step that must be followed to prevent and avoid any health risks. In addition, the lack of consensus regarding MRLs undermines pesticide controls, so the continuous, internationally harmonized monitoring of foods to ensure consumer safety is required. 

Over the past years, the main concern has been related to the potential risk of combined exposure to multiple pesticide residues in the diet and the dose addition of these compounds. According to the current regulations, the risk assessment of exposure to chemicals mainly relies on assessing individual substances and a few groups of substances that are expected to occur together [39]. The current methods used for human risk assessments assume that different components in mixtures act additively and behave as if they were dilutions of each other [39]. In this respect, the evaluation of exposure to multiple chemicals assumes that compounds with the same mechanism of toxicological action may have a cumulative effect that should be considered. In this regard, much effort has been put toward developing comprehensive frameworks dealing with human risk assessment of combined exposure to multiple chemicals [40,41]. As a result, methodologies developed enabled a grouping of chemicals into cumulative assessment groups (CAGs) based on their effects on target organs/systems and then with respect to their modes of action. Such methodologies have been developed only for multiple pesticide residues in food [42]. In 2021, EFSA published a report on a retrospective (2016–2018) cumulative risk assessment of dietary exposure to OPs (*n* = 36) and N-methyl carbamate insecticides (*n* = 11), which was conducted for chronic inhibition of erythrocyte acetylcholinesterase (AChE) [42]. It was concluded that cumulative exposure to pesticides, causing effects on the AChE, did not reach the threshold for regulatory consideration for any of the populations assessed [42].

However, the effects of combined exposure to multiple pesticide residues can be more complex due to their possible interactions. Scientific data about the possible synergistic effects of multiple pesticide residues as well as the effects of exposure to multiple residues that display different modes of action remains very limited [20,43]. Additionally, exposures to different chemicals may arise from separate sources, which should also be considered [44]. Consequently, these gaps in our knowledge may lead to an underestimation of the real health risk.

Recent nutritional recommendations to increase the consumption of fruit, vegetables, and whole grains may increase dietary pesticide intakes leading to severe cumulative toxicity and increased risk of various chronic illnesses, including cancer, respiratory, metabolic, reproductive, and neurologic disorders [1,45]. Urinary levels of pesticides or their metabolites are commonly used as biomarkers of human pesticide exposure [46]. Recently, the European Human Biomonitoring Initiative (HBM4EU) prioritized the collection of information on human exposure to pyrethroids pesticides, organophosphate pesticides (chlorpyrifos, dimethoate, and glyphosate), polyethoxylated tallow amine (additive in glyphosate formulations), and phenyl pyrazole insecticide (fipronil) for the period 2000–2022 [47]. However, as no proper urinary biomarkers existed for dimethoate and polyethoxylated tallow amine (POEA), the European human biomonitoring data on these substances was unavailable. The study results indicate extensive exposure to pyrethroids, chlorpyrifos, and glyphosate in the general European population, with noticeable geographical differences. The highest urinary levels for all the investigated pesticides were reported in Cyprus and Valencia (Spain) [47]. As for the OPs, the high detection rate of chlorpyrifos metabolite, 3,5,6-trichloro pyridine-2-phenol (TCP), was reported in most studies. However, as chlorpyrifos and chlorpyrifos-methyl have been banned in the EU since February 2020 [48], the exposure level in the general population is expected to have decreased. Recently, POEA exposure biomarkers have been identified; the first LC-MS/MS method for rapid analysis of 11 POEA homologues in human plasma was developed and validated using the plasma samples of glyphosate-poisoned patients [49]. 

Several studies have shown that organic food consumption may be one way to achieve a considerable reduction in dietary exposure to pesticides, including OPs, minimizing potential health risks [43,50,51,52]. Organic farming stipulates the non-use of synthetic fertilizers and most pesticides, leading to the absence or decrement of the concentration of pesticide residues in foods compared to conventional farming [45,52]. A recent study assessing the EU agricultural soils of organic and conventional farms reported that the pesticide residue levels in organic fields were 70–90% lower than in conventional ones [43]. However, although synthetic pesticides are not used in organic farming, pesticide residues can still be present in organic farming soils [53]. Furthermore, persistent compounds, such as DDT, remain at relatively high levels in organic fields, likely due to historical applications, despite being banned in many EU countries since the 1970s [54]. Therefore, to ensure minimal pesticide residue levels, transitioning to organic farming requires conversion transition periods adapted based on the initial residue mixtures and their residence time in the soil [43]. In addition, there is a severe research gap considering the effects of complex pesticide mixtures present in the soil-on-soil health and, consequently, on food quality and human health [43].

Several dietary intervention studies have shown that an organic diet significantly reduces urinary pesticide residue excretion compared to conventional food consumption [45]. However, these studies usually monitor a small number of selected pesticides and do not evaluate mineral- and plant-extract-based pesticides that are commonly used in organic farming. In addition, urinary pesticide residue excretion may result from both dietary and environmental pesticide exposure, and according to current knowledge, the relative contribution of these two sources to total chronic pesticide exposure is not possible to estimate [45].

The risk assessment of pesticide effects on human health and the environment is complex and considers the types and dosage of pesticides used, the periods and levels of exposure, and the environmental characteristics of the locality where pesticides are applied. In addition, although some toxic pesticides have been banned, they continue to be detected frequently in the environment due to their long degradation half-lives, thus contaminating the soil and water sources [55]. Therefore, although there is a requirement for pesticides to be produced, distributed, and used under regulations, due to their frequent applications, mistreatments, and heterogeneous regulatory limits, pesticides and their metabolites have been frequently detected in crops, agricultural soils, and water sources, posing a potential threat to human health [16,56]. Therefore, the cumulative risk assessment of the pesticide effects on human health should consider both the dietary and non-dietary routes of exposure and be regulated by an extensive legal framework harmonized globally to ensure and maintain food safety and security. Based on the above, developing and implementing improved strategies to protect human health and the environment is mandatory.

## 3. Strategies Aimed to Protect Human Health and the Environment from Pesticide Exposure

Due to the prominent scientific progress in chemistry, biology, and molecular biology, the approaches to protecting human health and the environment from pesticide exposure have continuously improved. These include searching for novel pesticides, developing methods for detecting and re-assessing the safety of the currently used pesticides, and developing methods for pesticide degradation into less toxic products. All of these approaches should meet the requirements of Integrated Pest Management (IPM), a strategy adopted by the EU in 2009 through Directive 2009/128/EC, also called the Sustainable Use of Pesticides Directive (SUD) [57]. The IPM strategy focuses on managing pests through a combination of sustainable biological, physical, and other non-chemical methods minimizing the risk to human health and the environment associated with the use of chemical products. According to IPM, chemical pesticides should be applied only as a last resort. Instead, the use of competitive plant material (e.g., resistant cultivars and certified seed), non-chemical tools (e.g., seed coating, flaming, beneficial microorganisms, etc.), and novel cultivation techniques (e.g., intercropping, crop rotation and diversification, stale seedbed technique, etc.) should be applied [58]. However, although this concept was made obligatory in the EU in 2014, limited progress has been achieved thus far, and goals set by the SUD have mainly been left unaccomplished [59].

General strategies for the development of new, effective, environmentally friendly pesticides encompass (i) the development of pesticides that are rapidly degradable and less residual in the environment; (ii) the development of pesticides that are effective at extremely low doses; and (iii) the development of selective chemicals effective in the control of pests but not toxic against humans or non-target species [60]. In the last decade, at least 105 pesticides (fungicides, insecticides, nematicides, acaricides, and herbicides) have been launched or are under development [60]. However, although most of them appear safe for humans and the environment, only a few products have been developed for practical use. Pesticide development has increasingly shifted from chemical to biological pesticides, including RNAi pesticides, abiotic stress control agents, genetically modified crops, and seeds, which are believed to affect the environment to a lesser extent than chemical pesticides [60]. It has been estimated that biological pesticides will equal chemical pesticides on the market by the 2050s [61]. Nevertheless, pesticides, including OPs, are frequently used and are expected to be continuously used in the future.

The detection of pesticides and re-assessment of the safety of the already-used pesticides envisage the development and implementation of new techniques with better reliability than existing ones. Novel techniques should enable a better prediction of the potential hazards of pesticides and, henceforward, contribute to reducing their adverse effects on human health and the environment. The simultaneous presence of different contaminants, including pesticides, in the same food (the so-called “cocktail effect”) represents a significant aspect of food safety that requires comprehensive research [62]. The possible interactions between different chemical contaminants in food may result in partial detoxification (if antagonism or inhibition takes place) or in an increase in toxicity due to synergism or potentiation, even when each compound is present at a level below toxicity reference values [44,63]. Therefore, there is an urgency to develop approaches that will enable the evaluation of these effects before confirming an effective risk [62]. Concerning the mixture risk assessment (MRA), according to Regulation (EC) No 396/2005 [9], the dietary risk assessment of pesticide exposure should take into account cumulative and synergistic effects in the setting of pesticide MRLs when methods become available. In 2021, the EU Commission and European Food Safety Authority (EFSA) developed and adopted an Action Plan (2022–2030) under document SANTE/10178/2021. It focuses on assessing human health risks from dietary and non-dietary exposure to pesticides and accelerates work on developing methods for cumulative risk assessment and their subsequent implementation [64]. As proposed, methodologies enabling human health risk assessment of combined exposure to multiple chemicals (RACEMiC) will be implemented by 2030 [39]. Currently, the methods, data, and tools for dietary MRA for pesticides are mostly available; however, significant scientific gaps considering the non-dietary exposure to pesticide mixtures still exist [39]. In addition, according to regulations, the dose-addition assumption will still be applied for the combined toxicity of the chemicals unless evidence for antagonistic or synergistic interactions is available [39]. Therefore, there is an urgency to increase knowledge about potential interactions among multiple contaminants in food to evaluate the effective risk of exposure. In this respect, the development of new analytical procedures and microbiological methods for food safety control is necessary [62].

Numerous detection methods based on chemical, physical and biological parameters have been utilized so far to identify even trace amounts of the pesticides. Traditional methods for pesticide detection comprise instrumental techniques such as gas chromatography (GC), high-performance liquid chromatography (HPLC), or chromatographic methods coupled with mass spectrometry (MS) detectors, which provide profuse qualitative and quantitative information on the residues with high accuracy [65]. However, the main limitations of these methods are the time-consuming sample preparation, the requirement for highly trained technicians, and the expensive equipment [66]. In that terms, Near-infrared spectroscopy (NIR), as a simple, reliable, and cheap technique, could be helpful as it can be used to predict soil composition and absorption of OPs, such as chlorpyrifos, methyl parathion, and phoxim [67]. In addition, significant research has been devoted to developing microfluidic paper-based analytical devices (µPADs) as an inexpensive alternative to highly sophisticated instrumentation in analytical applications for food and water monitoring that can be used for continual testing, especially in developing countries [68]. Furthermore, the use of µPAD sensors is frequently associated with smartphone-based detection of pesticides [67]. For example, Sicard et al. developed a highly selective and sensitive µPAD sensor and a mobile application suitable for on-site colorimetric identification of paraoxon and malathion based on the inhibition of immobilized AChE [69].

The development of sensors (electrochemical sensors, optical sensors, including chemiluminescence, fluorescence, and colorimetric sensors, and piezoelectric sensors) and biosensors represents a novel strategy for monitoring pesticide contamination of food. Moreover, introducing nanomaterials into their structure improves the efficacy of sensors and biosensors as analytical tools for detecting pesticides [66,67]. Using biosensors in pesticide detection might enable proficient and precise analysis at a low cost. AChE-based biosensors have been commonly used to detect diazinon, dimethoate, dichlorvos, chlorpyrifos, malathion, methyl parathion, glyphosphate, and other OPs [66]. However, the main limitation of this method is the lack of stability and persistent need for a substrate for quantifying the pesticide level. For that reason, several methods based on AChE inhibition, including colorimetric and electrochemical assays, have been developed to improve the system’s stability and enable a more efficient analysis with lower detection capabilities. Moreover, the immobilization of AChE with different nanocomposites has been considered a potent tool to increase the response of the biosensor in pesticide detection [70]. In that sense, numerous nanomaterials have been developed for detecting, degrading, and removing pesticides [65].

As discussed before, the food and feed might frequently be contaminated with more than one pesticide, so there is a growing need to develop and improve sensitive multi-residue detection methods. As comprehensively discussed by Jia et al. [71], two strategies for rapid multi-residue detection methods have been proposed; the first one, based on different recognition elements, comprises the use of antibodies, aptamers, and molecularly imprinted polymers (MIPs). The second strategy, based on the inherent characteristics of pesticides, encompasses the use of enzymatic inhibition-based sensors, NIR spectroscopy, and surface-enhanced Raman scattering (SERS) spectroscopy. In addition, numerous sensitive and reliable Liquid chromatography tandem mass spectrometry (LC-MS/MS) techniques and high-resolution mass spectrometry (LC-HRMS) techniques are developed and validated for simultaneous analysis of pesticides, veterinary drug residues, and other contaminants in foods [72,73,74]. Likewise, the method (CEN 15662) for pesticide residue analysis in foods proposed by European Committee for Standardization [75] encompasses a QuEChERS extraction followed by multi-residue determination on GC-MS/MS and LC-MS/MS. Furthermore, an advanced QuEChERS mega-method for simultaneous determination of at least 300 compounds, including pesticides, veterinary drugs, and environmental contaminants in matrices such as muscles of beef lamb, goat, and fish using LC-MS/MS and GC-MS/MS is also validated [76,77]. The proposed methods and their further improvements are expected to enable efficient and accurate multi-residue screening and detection.

## 4. Degradation Strategies for Organophosphate Pesticides

The broad-spectrum insecticidal activity, chemical stability, high efficiency, and low cost of production make OPs one of the predominant pesticides widely used in agriculture, veterinary practice, and urban areas. However, since they can seriously affect human health, their application has become one of the primary anthropogenic sources of environmental pollution. 

Numerous pesticides are not easily degradable; they remain in the soil, leak into groundwater and surface water, and contaminate the surrounding ecosystem. In addition, depending on their chemical qualities, they can enter the organism and bioaccumulate in food chains [78]. For this reason, efforts are being made to find effective ways for their degradation into non-toxic or less toxic forms to reduce their impact on humans and restore the pristine environment.

**Figure 2:**
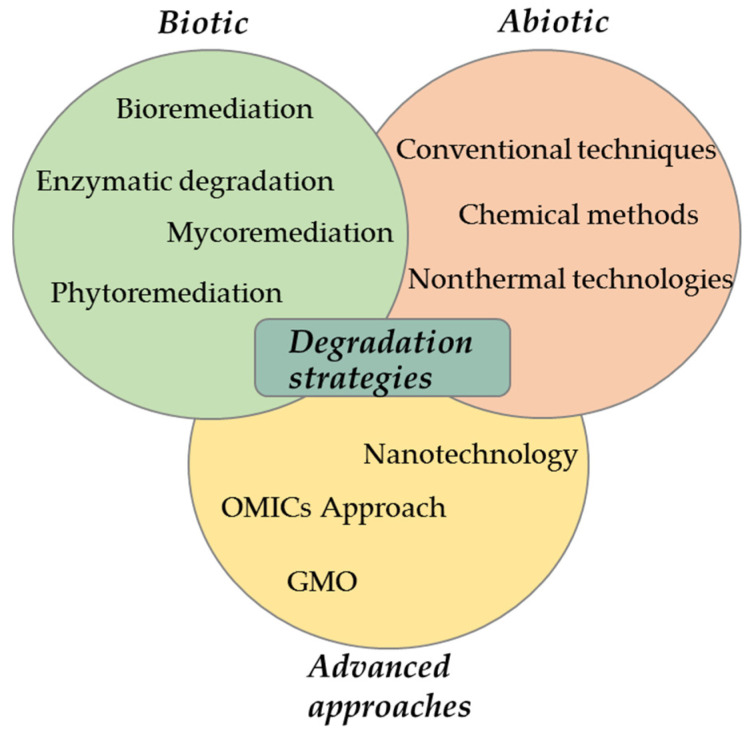
Biotic and abiotic degradation strategies applied for organophosphate pesticides.

Several biotic and abiotic degradation strategies have been developed and continuously improved to minimize human exposure to pesticides and their potential adverse environmental effects. Various biotic and abiotic techniques applied for OP degradation are presented in Figure 2. The biotic strategy includes transformation processes mediated by microorganisms, fungi, or plants, while the abiotic strategy implies the direct chemical or mechanical breakdown of pesticides into non-toxic forms. The types of transformation processes by which a pesticide will degrade depend on its structural affinity for specific types of transformation and the environmental conditions to which it is exposed [79].

### 4.1. Biotic Degradation Strategy

The biotic approach is based on the ability of microorganisms to convert hazardous contaminants into relatively simple and non-toxic compounds. Contaminants, including OPs, can be accumulated in the soil and agricultural runoff water through agricultural application, and their removal from the environment can be achieved by biodegradation and/or bioremediation. While biodegradation is a naturally occurring process, bioremediation is a man-made, engineered process whose efficiency may depend on moisture, temperature, redox conditions, organic matter, pH, and nutrients that influence chemical diffusion and microbial activity in the soil [80].

Biodegradation is a process that involves the complete decomposition of an organic compound into its inorganic constituents. Due to its low cost, simple application, high effectiveness, and lack of secondary contamination, biodegradation is considered an effective tool for the remediation of pesticide contamination [81,82,83]. Generally, under optimal conditions, biodegradation represents the bioconversion of a substance (via a series of intermediates) into small, inert end products (mineralization) [84]. Advances in approaches for soil pesticide degradation, such as biostimulation and bioaugmentation, may enable effective OP detoxification. In contrast to bioattenuation, which occurs naturally without human intervention, biostimulation enables accelerated biodegradation by providing the right conditions for microorganisms in the soil. The optimum nutritional ratio of carbon, nitrogen, and phosphorus is crucial for biostimulation [85]. Land farming and composting are biostimulation activities that involve carbon sources, nutrients, and humidity control [86].

Nowadays, the remediation industry and scientific community are focusing on bioremediation systems that use bioaugmentation processes. Altered microorganisms, obtained from the environment or genetically modified in the laboratory, are often utilized in bioaugmentation to accelerate the detoxification and breakdown processes in contaminated environments [86]. However, bioremediation is restricted to biodegradable compounds since not all toxins in contaminated soils are substrates for microbial absorption.

In general, the biological removal of organophosphorus compounds has become the method of choice since many microorganisms have been found to have the metabolic pathways and enzymes necessary for the degradation of a variety of xenobiotic compounds, including OPs [87]. Microorganisms are the most significant candidates for biodegradation/bioremediation because of their straightforward cellular structure, small genome size, quick replication, rapid evolution, and adaptation to contaminated environments [88]. In addition, microorganisms can metabolize contaminants, including OPs, using them as nutrient and/or energy sources. Bacteria, fungi, actinomycetes, and algae are the most capable bio-transformers and pesticide degraders [84,89].

A wide range of microorganisms is reported to selectively hydrolyze a variety of organophosphorus contaminants, including the species of the genera *Arthrobacter* [90], *Enterobacter* [91], *Burkholderia* [90], *Pseudomonas* [92], *Serratia* [93], *Sphingobium* [87] and *Bacillus*, *Flavobacterium*, *Micrococcus*, and *Plesiomonas* [94], algae such as *Chlorella, Stichococcus*, and *Scenedesmus* [95,96], as well as fungi including *Penicillium oxalicum* [97], *Fusarium* sp. [98] and *Aspergillus sydowii* [99], *Cladosporium cladosporioides* [100], *Aspergillus niger* [101], *Aspergillus fumigates* [102], among others. 

In 1973 the first bacteria, *Flavobacterium* sp., capable of degradation of organophosphorus compounds was discovered [103]. *Flavobacterium* can degrade almost all known P-O bonds through enzymatic hydrolysis. Next, the bacteria *Pseudomonas diminuta*, which acts similarly by cleaving P-O bonds by OP-degrading enzymes, was isolated in the United States in 1982 [104]. Many bacteria and fungi that may utilize organophosphorus compounds as a carbon, nitrogen, or phosphorus source have been discovered in subsequent years. 

The microbial degradation of OPs involves complex processes of oxidation, reduction, hydrolysis, dealkylation, hydroxylation, alkylation, and ring cleavage, where hydrolysis usually represents the first stage in the degradation process, followed by continued degradation of the less hazardous compounds [105,106]. Usually, phosphorus is present as a phosphonate or a phosphate ester. As esters, they possess several hydrolysis-vulnerable sites. Microorganisms degrade OPs by hydrolyzing P-O alkyl and aryl bonds, which is a crucial step in detoxification [94,105,107]. The degradation rate varies and depends on microorganism species, their catalytic activities, and various environmental parameters such as temperature, pH, and sunlight availability [108]. In general, the action of bacteria is related to their genes and associated enzymes that hydrolyze and detoxify OPs [94]. Numerous functional genes have been reported to date, including *opd* (*opdE, opdA, opdC*), *amp* (*ampA*), *oph* (*ophB*), and *mpd* [109,110], while enzymes involved in the biodegradation and detoxification of OPs include cytochromes P450, phosphatase, esterase, hydrolase, and oxygenase [111]. Most studied OP degrading enzymes are hydrolases, such as organophosphorus hydrolase (OPH), organophosphorus acid anhydrolase (OPAA), and methyl parathion hydrolase (MPH) [105]. 

The OPHs, also termed phosphotriesterases and paraoxonase, are found in microorganisms, animals, and plants and are the most widely studied OP-degrading enzymes due to their catalytic efficiency [106]. OPHs are metalloenzymes that hydrolyze the triester linkage in organophosphate insecticides. These enzymes are encoded by the OP-degrading (*opd*) gene and have been initially found in *Sphingobium fuliginis* (*Flavobacterium* sp.) and *Brevundimonas diminuta* (*Pseudomonas diminuta*) [103,112]. The amino acid sequences of these enzymes are highly consistent; they share the same (α/β)_8_-barrel structural folds and an active site with two transition metal ions, such as zinc, iron, cobalt, or manganese [113]. Although the natural substrate of OPH remains unclear, it was verified that synthetic paraoxon is the best substrate for OPH. Additionally, OPH effectively hydrolyzes organophosphate triglyceride pesticides containing P-O bonds, such as paraoxon, parathion, and diazinon, but also hydrolyzes P-F, P-CN, and P-S bonds [114]. In addition, a variant of the OPH enzyme, OPDA encoded by the *opdA* gene, obtained from *Agrobacterium radiobacter* [115], can hydrolyze a wide range of OPs and G-type nerve agents like tabun, sarin, soman, and ethylsarine [116]. Moreover, OPDA is the only enzyme currently used commercially for bioremediation and pesticide decontamination of water sources [117,118]. 

OP-degrading enzyme OPAA, encoded by the *opaA* gene, was identified in *Alteromonas undina* and *Alteromonas haloplanktis* [119] as a member of the dipeptidase family with no enzyme or gene-sequence similarities with OPH or MPH. It hydrolyzes OPs and nerve agents G/V-series, acting on the P-O, P-F, P-S, and P-CN bonds [120]. 

Numerous methyl parathion degrading (*mpd*) genes were cloned to date, and phylogenetic analysis showed that they evolved apart from *opd* genes. The analysis also suggested that *mpd* and *β-lactamase* gene homologs, both members of the β-lactamase superfamily, are present in *Methylibium petroleiphilum, Azoarcus* sp., *Leptothrix cholodnii, Chromobacterium violaceum* and *Sinorhizobium meliloti* [121].

Different fungi are also involved in the remediation of contaminants in wastewater, soil, and organic wastes. The elimination of contaminants, including pesticides, through the utilization of fungi is known as mycoremediation [122,123]. Toxins and contaminants can be stored within fungal structures and serve as a carbon source upon enzymatic degradation [124]. In general, fungi have shown the potential to transform or degrade harmful pesticides into non-harmful or less harmful compounds through oxidation, decarboxylation, and enzymatic hydrolysis. Numerous fungi, including *Penicillium spp., Fusarium spp., Aspergillus flavus*, *Aspergillus niger*, *Trichoderma harzianum, Trametes versicolor, Pleurotus ostreatus, Lentinula edodes, Bjerkandera adusta*, *Rhizoctonia solani*, *Sporothrix cyanescens*, *Mortierella* are involved in this process [125,126,127,128]. Studies have shown that *Aspergillus oryzae*, *Aspergillus niger*, *Aspergillus flavus*, *Penicillium waksmanii*, *Acremonium* sp. participate in decomposing OPs, such as chlorpyrifos, malathion, parathion, and ethion [128,129]. For example, *Aspergillus oryzae* degrades malathion into β-monoacid and dicarboxylic acid by carboxylesterase activity, successively converting it into inorganic phosphate. Furthermore, *Candida cylindracea* and *Fusarium oxysporum* are also reported to degrade malathion by hydrolyses [130]. The *Penicillium waksmanii* is shown to degrade parathion into aminoparathion, which is less hazardous than the parental compounds [129]. Similarly, *Aspergillus flavus*, *Aspergillus niger*, and *Trichoderma harzianum* are found to degrade chlorpyrifos and endosulfan [127,128]. 

In general, mycoremediation can be considered a cost-effective and eco-friendly approach for the degradation of pesticides since fungi grow and survive in diverse agroecosystems. Also, numerous fungi have shown the potential to transform or degrade harmful pesticides into non-harmful or less harmful compounds. Therefore, mycoremediation is extremely advantageous in protecting the environment and living organisms, including humans.

Phytoremediation, also known as plant-assisted bioremediation, is a solar-powered technology that uses contaminant-scavenging plant species [131]. During this process, plants remove contaminants, including pesticides, from the environment through phytoextraction, phytodegradation, phytovolatilization, and rhizofiltration [132,133,134] and transform them into less dangerous ones [135]. The major plant-associated enzymes involved in pesticide phytoremediation are carboxylesterase, cytochrome P450, and glutathione S-transferase [136,137]. Although phytoremediation is a cost-effective and valuable method for remediation, it has certain restrictions, such as the requirement for contaminants to be in the zone accessible for plant roots [138]. Furthermore, if pesticides are highly water soluble, the root system will not be able to reach them, and no degradation will occur. In addition, excessive pesticide concentrations can be hazardous to plants [123]. 

### 4.2. Abiotic Degradation Strategy

The abiotic strategy encompasses various approaches applied to reduce pesticide residue levels in foods and feeds to maximum permissible limits (MRLs) as prescribed by regulatory bodies, thus minimizing the risk of consumer exposure. However, there are many challenges in these processes, especially in terms of detecting the metabolites or intermediates of the pesticide degradation, which in some cases could be more toxic than the parent compounds. The complexity of the compounds present in the food matrix, the identification of numerous degradation products that may be formed by different pathways that are difficult to predict, and the lack of adequate commercial standards for the degraded products are just some of the challenges that occurred during the assessment of the toxicity of the pesticide degradation products [139]. As a consequence, numerous studies investigating pesticide reduction using different technologies have not implemented the screening and detection of degraded pesticide metabolites.

As shown in Figure 2, conventional techniques (chemical washing, peeling, drying, heating, etc.) and advanced approaches, such as chemical and nonthermal methods, have been employed in food processing to degrade pesticide residues.

Conventional methods, such as washing and cleaning, are of limited efficacy in pesticide removal due to the hydrophobicity of numerous pesticides. On the other hand, heat processing techniques, such as saucing, canning, blanching, and boiling, may significantly (but often not completely) reduce pesticide levels [31,140,141]. However, these techniques may be followed by a reduction of nutritional and taste-related features of foods and are not appropriate for vegetables and fruits that are consumed raw [142]. 

The chemical methods for the oxidative degradation of OPs mainly include the utilization of the various chemical oxidants, such as ferrate (VI), manganese dioxide, manganese dioxide charged with bisulfite, and nano-structured titania-iron mixed oxides, which provided encouraging results in environmental remediation, especially treatments of water [143,144,145,146]. However, limited effects of chemical techniques on pesticide removal rates are also reported [147]. 

The nonthermal technologies (cold plasma, high pressure processing, pulsed electric field, ultrasound, pulsed light, ultraviolet light, irradiation, oscillating magnetic field, ozonization, etc.) were developed to overcome the disadvantages of the conventional methods and to facilitate pesticide residue removal in fresh fruits and vegetable products and the environment as well [147,148]. 

As a feasible and relatively cheap technique, ozonated water washing has been frequently used for OPs (methyl-parathion, parathion, diazinon, chlorpyrifos) degradation in fruits and vegetables [142]. However, it was demonstrated that degradation products of pesticide residues upon ozone treatment (methyl paraoxon, paraoxon, and diazoxon) were more toxic than the parent compounds, which indicates the need for further processing using different technologies. In addition, this process requires higher treatment time and enhances the risk of oxidative degradation of bioactive substances in food commodities [149]. 

Gamma irradiation has also been used efficiently for the degradation of chlorfenvinphos, dimethoate, diazinon, and profenofos in the environment, especially in water [150]. Moreover, the extended treatment of chlorfenvinphos in tap water and groundwater further removed its degradation products [151]. However, these techniques are less feasible in food processing, especially in pesticide removal from rough-surfaced foods [142]. 

Ultrasonication is an unexplored area for pesticide removal from food commodities. This method was commonly performed in combination with ozone and UV irradiation treatments, enhancing their efficacy [142]. Recently, a novel advanced oxidation process (AOP), i.e., the coupled free chlorine/ultrasound (FC/US) process, was utilized for the removal of dimethoate, trichlorfon, and carbofuran from lettuce, where removal efficiencies reached 86.7%, 79.8%, and 71.3%, respectively [152]. No noticeable damage to the quality of vegetables was observed after the FC/US process. However, when used solely, the efficacy of ultrasonic washing in pesticide degradation was variable and dependent on the surface morphology of the food commodities, with a variable impact on their nutritional properties [153].

High-pressure processing (HPP) is an environmentally friendly technology proposed as useful in reducing food contaminants such as pesticides and mycotoxins [154]. The efficiency of the HPP process depends on processing parameters, the chemical structure of the pesticide or mycotoxin, and the food matrix [154]. For example, its application successfully reduced chlorpyrifos levels in tomato samples under optimized conditions [155].

Cold plasma has been investigated for a wide range of applications, including its potential use for pesticide removal from agricultural commodities and wastewater [156]. The efficiency of plasma for pesticide reduction depends on several factors, such as the type of gas used in plasma and its flow rate, electrode distance, plasma power or voltage, exposure time, and others. These factors determine the amount of active species available for the pesticide reaction and subsequent degradation [142]. In the case of OPs, it has been shown that plasma species supplant the phosphoryl groups of pesticides, forming the phosphoxons, the unstable, toxic metabolites of the parent compound. It was also demonstrated that the toxicity of the formed metabolites largely depends on the chemical structure of the pesticides. A study analyzing the reduction of omethoate and dichlorvos in goji berry after gas barrier discharge plasma treatment reported increased toxicity of the products upon initial plasma treatment time of 0–6 min, after which it declined [157]. The study investigating chlorpyrifos degradation in tomatoes showed an 89.18% reduction after the 5 W plasma power for 6 min treatment [158]. The detection of the secondary metabolite, TCP, after the plasma treatment was also confirmed, but it was shown to be less toxic compared to the parent chlorpyrifos and its primary metabolite, chlorpyrifos-oxon. In addition, further intensified treatments led to the complete mineralization of the TCP metabolite [158]. Similar effects were reported upon plasma degradation of diazinon and phoxim in cucumber and table grapes, respectively [159,160]. Even though an encouraging rate of pesticide reduction was observed in the mentioned studies, some adverse effects, such as changes in the texture and total phenolic content of commodities, were also observed [158].

The pulsed electric field (PEF) methodology has been widely used for food preservation, reducing food contaminants, maintaining the nutritional values of the products, and removing pesticide residues from foods and wastewater [161]. For example, a study investigating the degradation of 16 pesticide residues in raw strawberries after treatment with PEF and boiling reported a removal efficacy of 92.9% and up to 91.2% when combined with ultrasonication [162]. A similar removal efficiency was reported for pyraclostrobin, chlorpyrifos ethyl, cyprodinil, malathion, and tau-fluvalinate in cherry juice after treatment with PEF (24.7 kV/cm, 655 μs) in combination with ozone and ultrasonication [163]. Nevertheless, additional studies on various food commodities are needed to support these findings. 

In recent years, extensive research has been dedicated to the effects of UV light irradiation on the pesticide residues retained in fruits and vegetables and the environment. It was shown that pesticide degradation by photolysis depends on the type of pesticide residues, UV light sources, light intensity, and irradiation time [164]. Some previous studies analyzing photodegradation of OPs in the honey, including coumaphos, methyl parathion, and fenitrothion after 1 h with 250, 500, and 750 W/m^2^ sunlight irradiation, have shown that coumaphos exhibited the best degradation performance (97.02% after 1 h) under 750 W/m^2^ sunlight irradiation [165]. Recently, numerous studies have been conducted to investigate the degradation of pesticides using vacuum ultraviolet (V-UV) and ultraviolet light-C (UV-C) light sources. It was shown that V-UV was more effective than UV-C in the degradation of some fungicides under the same reaction conditions [166]. Likewise, it was reported that V-UV/UV used to remove some carbamate pesticides from the water was much more effective than UV, reaching the removal efficiency of at least 90% at a V-UV fluence of 12 mJ/cm^2^ [167]. As most pesticides show absorption maxima at relatively short UV wavelengths, their photostability under the UV-C treatment and the toxicity of their photodegradation products should be assessed. A recent study investigating the toxicity of chlorpyrifos and its formulations (emulsifiable concentrate—EC and oil-in-water emulsion—EW) after UV-C irradiation showed that chlorpyrifos concentration decreased during UV-C irradiation. In contrast, the concentration of its product, chlorpyrifos-oxon, increased, reaching a maximal concentration after 17 min (EW) and 80 min (chlorpyrifos and EC) of irradiation, when subsequently decreased [168]. The same study demonstrated the pro-oxidative and genotoxic effects of their photodegradation products. Noteworthy, chlorpyrifos was more genotoxic compared to its formulations. Another study investigating the effects of UV-C irradiation of glyphosate in water showed at least  a 90% reduction in glyphosate concentrations and the generation of less toxic degradation products, reducing the overall toxicity to aquatic organisms [169]. Several techniques using UV have been applied for pesticide removal from wastewater. For example, a study using pulsed light (PL) technology for the photodegradation of several OPs in water showed >50% pesticide removal in a very short time [170]. However, the toxicity of photodegradation products was not assessed. 

Catalyst methodologies have also been used for pesticide removal, mainly for wastewater treatment and environmental remediation [171]. The photocatalytic process is based on pesticide residue breakdown by AOP, where photons degrade pesticides to CO_2_, H_2_O, and inorganic compounds. Usually, it comprises a catalyst such as TiO_2_, which, combined with UV light, accelerates the degradation of pesticides, mainly in the soil and agricultural wastewater [171,172]. For example, UV-C/TiO_2_ was efficiently used for the photocatalytic degradation of diazinon in water [173]. A similar photocatalytic activity was also reported for the CoFe_2_O_4_@TiO_2_ nanocomposite used to degrade chlorpyrifos [174]. An interesting study showed that the rate of degradation of malathion by UV light alone was lower than that observed when photocatalytic treatments, such as UV/H_2_O_2_, UV/TiO_2_, and UV/Fenton systems, were applied; however, in contrast to the photocatalytic processes, no increase in toxicity of the malathion aqueous solution after UV irradiation alone was observed [175]. Therefore, it is assumed that applications of photocatalytic oxidation in food processing can be limited due to the observed toxicity of the treated solutions [147].

In general, identifying pesticide degradation products and assessing their toxicity while employing different pesticide removal methodologies have to be carried out in order to ensure food safety and, thus, protect human health.

## 5. Future Perspectives on Pesticide Use and Management 

One of the biggest challenges in the 21st century is how to feed the increasing population while decreasing the adverse consequences on the environment and human health that arose due to the continuous deployment of pesticides, fertilizers, and freshwater [176,177]. The European Green Deal and its Farm to Fork Strategy proposed targets to establish sustainable agriculture, considering nature conservation to ensure a fair, healthy, and environmentally friendly food system [178]. Under the Farm to Fork Strategy [178], the EC plans to take actions to reduce by 50% the use and risk of chemical pesticides, including the use of more hazardous pesticides, until 2030. To achieve this goal, the EC will revise the SUD [57] and promote alternative practices, such as IPM, to protect harvests from pests and diseases. In this respect, strong support should be given to farmers and accelerate the transition toward sustainable agriculture. In addition, the Farm to Fork Strategy encourages organic farming intending to have at least 25% of EU agricultural land under organic farming management by 2030 [178]. However, some issues must be resolved to meet the proposed targets. As discussed, the risk assessment considering pesticide cocktails found in the major agricultural systems must be implemented since their effects on soil health and, consequently, on food safety are still unknown [43]. In addition, the issue of legacy pesticides that can persist in the environment for several decades after they were banned must be resolved [179]. Since current EU policy [178] leaves these issues unaddressed, improved strategies encompassing innovative methods to test the effects of pesticide cocktails on soil health and targeting and remediation of legacy pesticides in the environment are urgently required [43,179]. Soil remediation as well as the establishment of rich above-ground plant systems, should be prioritized to alleviate the effects of current and legacy pesticides in soils [43]. Thus, besides reducing pesticide usage, to ensure food safety, it is necessary to implement novel approaches to detect pesticide residues, assess the real risk of combined exposure to multiple residues, and degrade them into non-toxic products to safeguard consumer health. 

Considering pesticide screening and detection, the new procedures developed within the green chemistry framework should be prioritized [63]. In this regard, novel analytical techniques have been proposed based on nanosystems for accurate, green, and ultrasensitive detection of pesticide residues in food and the environment [180,181]. In addition, novel, smartly engineered nanomaterials, and advanced instrumentation should facilitate pesticide detection in complex food matrices and make it more sensitive, cost-effective, and less time-consuming [180].

Similarly, pesticide degradation strategies should rely on efficient, cost-effective, environmentally friendly techniques. The integration of nanotechnology and advanced materials can offer innovative approaches to pesticide degradation. Nanomaterials can be designed to enhance the efficiency of degradation processes through increased surface area, catalytic activity, and selectivity [179]. Moreover, green nanomaterials, produced using different parts of plants (seeds, fruit, leaves, and flower) or microorganisms (bacteria, algae, and fungi), are suggested to be biocompatible, biodegradable, cost-effective, eco-friendly, and efficient in environmental remediation [182]. However, there is a severe knowledge gap concerning identifying metabolites or intermediates of pesticide degradation, which in some cases could be more toxic than parental compounds. Therefore, further comprehensive studies on pesticide degradation products and their toxicity are required. 

Another promising direction in pesticide degradation is developing and implementing advanced, more efficient bioremediation techniques using various bioinformatics tools [183]. Genome engineering by gene editing tools, such as CRISPR-Cas, ZFN, and TALEN, can create functionally improved microorganisms with complex genes that encode catabolic enzymes involved in OPs metabolism [183,184]. However, releasing genetically engineered organisms into the environment requires the approval of various regulatory bodies. So, the direct application of recombinant enzymes, frequently termed cell-free catalytic systems derived from engineered microbes, which are non-replicative, can be used for environmental remediation [185].

The new postgenomic research technologies, called the OMICs approach, may provide tools to investigate microbial interactions with pesticides and construct enzyme-based mechanisms for bioremediation in different environmental settings [185]. The bioinformatics and computational tools in OMICs comprise technologies such as metagenomics, transcriptomics, proteomics, and metabolomics, as well as studies of their interactive pathways, named interactomics [186]. Integration of these technologies creates a multi-omic approach that provides a comprehensive understanding of the processes associated with biodegradation [185]. In this respect, it has been reported that CRISPR-Cas, ZFN, and TALEN as gene editing tools utilizing Pseudomonas, Escherichia coli, and Achromobacter sp. can be employed for remediation of chlorpyrifos, methyl-parathion, carbaryl, triphenyltin, and triazophos by constructing a guide RNA (gRNA) for expressing specific genes for the bioremediation [187]. In addition, computational analysis such as molecular docking, molecular modeling, and simulation analysis can efficiently determine the fate of degraded metabolites, the structural and functional characterization of OPs degrading enzymes, and their binding properties [188]. Developing an artificial microbiome with functionally specific species has also been proposed to facilitate bioremediation processes [183]. There are some concerns that without innovations, such as the New Genomic Techniques (NGT), the encouragement of gene-editing research, and the revision of the current EU legislation concerning genetically modified organisms (GMO) [189], the targets proposed by the Farm to Fork Strategies will be difficult to reach. Additional research in this area and more in-depth practical implementation of the techniques mentioned in large-scale studies are necessary.

Concerning the use of bioremediation techniques in food processing, it was demonstrated that some fermented foods could be detoxified from pesticides due to the activity of the bacterial microflora [190]. The lactic acid bacteria existing in or added to foods can metabolize a wide range of OPs, using them as a source of carbon and energy [191]. Fermentation by natural microflora or by probiotic strains added to foods is a promising approach for pesticide detoxication. However, since the exact metabolic pathways of degradation are still unknown, further research is necessary [190].

To ensure the development of sustainable agriculture, smart agricultural research employing different artificial intelligence (AI) approaches, such as deep learning (DL), machine learning (ML), agricultural robots, and robotics, has been suggested to resolve the prevailing problems in agriculture and improve productivity [192,193]. Using robotics can increase production and save time on repetitive tasks. It is estimated that low-cost agricultural robots can reduce pesticide usage by 80% if farmers use them for spraying [192,194]. Application of neural networks, DL, and ML techniques can enable early and timely identification of pests and diseases, monitoring of moisture and nitrogen content in the soil, informing irrigation for water saving, detection of herbicide usage, detecting food damage, etc. [193,195,196]. The proposed methods need to be further explored to establish the AI framework that should enable the sustainable development of smart agriculture.

Altogether, to develop a sustainable agriculture and food system, the whole environment in which food is produced should be considered. Besides taking appropriate agricultural management measures, it is necessary to improve and globally harmonize methodologies in the areas of food safety and food quality to protect human health. A holistic approach covering the entire food production chain should be applied to control food contaminants. In addition, the food-producing systems from farm to fork are influenced by several factors, such as climate change, demographics, and the economy, which may create new food safety risks and affect human health [197]. Collaborative efforts among scientists, policymakers, regulators, industry stakeholders, and farmers are crucial to closing the gaps between the EU strategy goals and practical implementations. Embracing innovative technologies, promoting sustainable practices, and fostering knowledge exchange on a global level are required to establish sustainable agriculture and ensure a fair and sustainable food system that does not leave anyone behind.

## Data Availability

Data is contained within the article.

## References

[1] Onyeka Kingsley N., Ayibapreye J., Ramón Eduardo Rebolledo R. (2022). Chemical Pesticides and Food Safety. Insecticides.

[2] Antonini C., Argilés-Bosch J.M. (2017). Productivity and environmental costs from intensification of farming. A panel data analysis across EU regions. J. Clean. Prod..

[3] Rajmohan K.S., Chandrasekaran R., Varjani S. (2020). A Review on Occurrence of Pesticides in Environment and Current Technologies for Their Remediation and Management. Indian J. Microbiol..

[4] Food and Agriculture Organization of the United Nations (FAO) (2022). FAOSTAT Database. Pesticides Use. http://www.fao.org/faostat/en/#data/RP.

[5] Donley N. (2019). The USA lags behind other agricultural nations in banning harmful pesticides. Environ. Health.

[6] Ambrus Á., Szenczi-Cseh J., Doan V.V.N., Vásárhelyi A. (2023). Evaluation of Monitoring Data in Foods. Agrochemicals.

[7] Sarkar S., Dias J., Gil B., Keeley J., Möhring N., Jansen K., European Parliament, Directorate-General for External Policies of the Union (2021). The Use of Pesticides in Developing Countries and Their Impact on Health and the Right to Food, European Parliament. https://data.europa.eu/doi/10.2861/28995.

[8] Food and Agriculture Organization of the United Nations (FAO), World Health Organization (WHO) (2020). Codex Alimentarius, Pesticides.

[9] (2005). Regulation (EC) No 396/2005 of the European Parliament and of the Council of 23 February 2005 on Maximum Residue Levels of Pesticides in or on Food and Feed of Plant and Animal Origin and Amending Council Directive 91/414/EEC Official Journal of the European Union, 70; pp. 1–16. https://eur-lex.europa.eu/legal-content/EN/TXT/?uri=CELEX:02005R0396-20210525.

[10] US Environmental Protection Agency (2002). Summary of the Federal Food, Drug, and Cosmetic.

[11] CIRS China. https://www.cirs-group.com/en.

[12] Agência Nacional de Vigilância Sanitária—Anvisa. https://www.gov.br/anvisa/pt-br/english/regulation-of-products/pesticides.

[13] van den Dries M.A., Lamballais S., El Marroun H., Pronk A., Spaan S., Ferguson K.K., Longnecker M.P., Tiemeier H., Guxens M. (2020). Prenatal exposure to organophosphate pesticides and brain morphology and white matter microstructure in preadolescents. Environ. Res..

[14] Zikankuba V.L., Mwanyika G., Ntwenya J.E., James A. (2019). Pesticide regulations and their malpractice implications on food and environment safety. Cogent Food Agric..

[15] Kwong T.C. (2002). Organophosphate Pesticides: Biochemistry and Clinical Toxicology. Ther. Drug Monit..

[16] Fu H., Tan P., Wang R., Li S., Liu H., Yang Y., Wu Z. (2022). Advances in organophosphorus pesticides pollution: Current status and challenges in ecotoxicological, sustainable agriculture, and degradation strategies. J. Hazard. Mater..

[17] Kim K.H., Kabir E., Jahan S.A. (2017). Exposure to pesticides and the associated human health effects. Sci. Total Environ..

[18] Leskovac A., Lazarević-Pašti T. (2022). Organophosphate Pesticides: Cytotoxicity, Genotoxicity and Current Treatment Strategies. Organophosphates: Detection, Exposure and Occurrence. Volume 1: Impact on Health and the Natural Environment.

[19] Petrovic S., Lazarević-Pašti T. (2022). Organophosphate Pesticides and Human Health: Current Knowledge and Future Prospects. Organophosphates: Detection, Exposure and Occurrence. Volume 1: Impact on Health and the Natural Environment.

[20] Kubiak-Hardiman P., Haughey S.A., Meneely J., Miller S., Banerjee K., Elliott C.T. (2022). Identifying Gaps and Challenges in Global Pesticide Legislation that Impact the Protection of Consumer Health: Rice as a Case Study. Expo. Health.

[21] Hejazi M., Grant J.H., Peterson E. (2022). Trade impact of maximum residue limits in fresh fruits and vegetables. Food Policy.

[22] Jors E., Neupane D., London L. (2018). Pesticide Poisonings in Low- and Middle-Income Countries. Environ. Health Insights.

[23] Kenya, Pesticides Atlas (2022). Data and Facts on Toxins in Agriculture.

[24] Wolejko E., Lozowicka B., Jablonska-Trypuc A., Pietruszynska M., Wydro U. (2022). Chlorpyrifos Occurrence and Toxicological Risk Assessment: A Review. Int. J. Environ. Res. Public Health.

[25] Foong S.Y., Ma N.L., Lam S.S., Peng W., Low F., Lee B.H.K., Alstrup A.K.O., Sonne C. (2020). A recent global review of hazardous chlorpyrifos pesticide in fruit and vegetables: Prevalence, remediation and actions needed. J. Hazard. Mater..

[26] Lopez-Carmen V.A., Erickson T.B., Escobar Z., Jensen A., Cronin A.E., Nolen L.T., Moreno M., Stewart A.M. (2022). United States and United Nations pesticide policies: Environmental violence against the Yaqui indigenous nation. Lancet Reg. Health Am..

[27] Akomea-Frempong S., Ofosu I.W., Owusu-Ansah E.d.-G.J., Darko G. (2017). Health risks due to consumption of pesticides in ready-to-eat vegetables (salads) in Kumasi, Ghana. Int. J. Food Contam..

[28] Ssemugabo C., Bradman A., Ssempebwa J.C., Sille F., Guwatudde D. (2022). Pesticide Residues in Fresh Fruit and Vegetables from Farm to Fork in the Kampala Metropolitan Area, Uganda. Environ. Health Insights.

[29] Gill J.P.S., Bedi J.S., Singh R., Fairoze M.N., Hazarika R.A., Gaurav A., Satpathy S.K., Chauhan A.S., Lindahl J., Grace D. (2020). Pesticide Residues in Peri-Urban Bovine Milk from India and Risk Assessment: A Multicenter Study. Sci. Rep..

[30] El-Sheikh E.A., Ramadan M.M., El-Sobki A.E., Shalaby A.A., McCoy M.R., Hamed I.A., Ashour M.B., Hammock B.D. (2022). Pesticide Residues in Vegetables and Fruits from Farmer Markets and Associated Dietary Risks. Molecules.

[31] El-Sheikh E.-S.A., Li D., Hamed I., Ashour M.-B., Hammock B.D. (2023). Residue Analysis and Risk Exposure Assessment of Multiple Pesticides in Tomato and Strawberry and Their Products from Markets. Foods.

[32] Bouhala A., Lahmar H., Benamira M., Moussi A., Trari M. (2020). Photodegradation of Organophosphorus Pesticides in Honey Medium by Solar Light Irradiation. Bull. Environ. Contam. Toxicol..

[33] EFSA (European Food Safety Authority) (2018). The 2016 European Union report on pesticide residues in food. EFSA J..

[34] Witczak A., Pohoryło A., Abdel-Gawad H., Cybulski J. (2018). Residues of some organophosphorus pesticides on and in fruits and vegetables available in Poland, an assessment based on the European union regulations and health assessment for human populations. Phosphorus Sulfur Silicon Relat. Elem..

[35] Mert A., Qi A., Bygrave A., Stotz H.U. (2022). Trends of pesticide residues in foods imported to the United Kingdom from 2000 to 2020. Food Control..

[36] Panseri S., Bonerba E., Nobile M., Di Cesare F., Mosconi G., Cecati F., Arioli F., Tantillo G., Chiesa L. (2020). Pesticides and Environmental Contaminants in Organic Honeys According to Their Different Productive Areas toward Food Safety Protection. Foods.

[37] Nardelli V., D’Amico V., Ingegno M., Della Rovere I., Iammarino M., Casamassima F., Calitri A., Nardiello D., Li D., Quinto M. (2021). Pesticides Contamination of Cereals and Legumes: Monitoring of Samples Marketed in Italy as a Contribution to Risk Assessment. Appl. Sci..

[38] Carrasco Cabrera L., Di Piazza G., Dujardin B., Medina Pastor P., EFSA (European Food Safety Authority) (2023). The 2021 European Union report on pesticide residues in food. EFSA J..

[39] de Jong E., van der Voet H., Marx-Stoelting P., Bennekou S.H., Sprong C., Bloch D., Burchardt A., Lasch A., Opialla T., Rotter S. (2022). Roadmap for action on Risk Assessment of Combined Exposure to Multiple Chemicals (RACEMiC). EFSA Support. Publ..

[40] EFSA (European Food Safety Authority) (2013). International framework dealing with human risk assessment of combined exposure to multiple chemicals. EFSA J..

[41] EFSA (European Food Safety Authority) (2019). Guidance on harmonised methodologies for human health, animal health and ecological risk assessment of combined exposure to multiple chemicals. EFSA J..

[42] Anastassiadou M., Choi J., Coja T., Dujardin B., Hart A., Hernandez-Jerrez A.F., Jarrah S., Lostia A., Machera K., EFSA (European Food Safety Authority) (2021). Mohimont Cumulative dietary risk assessment of chronic acetylcholinesterase inhibition by residues of pesticides 2021. EFSA J..

[43] Geissen V., Silva V., Lwanga E.H., Beriot N., Oostindie K., Bin Z., Pyne E., Busink S., Zomer P., Mol H. (2021). Cocktails of pesticide residues in conventional and organic farming systems in EuropeLegacy of the past and turning point for the future. Environ. Pollut..

[44] Cattaneo I., Kalian A.D., Di Nicola M.R., Dujardin B., Levorato S., Mohimont L., Nathanail A.V., Carnessechi E., Astuto M.C., Tarazona J.V. (2023). Risk Assessment of Combined Exposure to Multiple Chemicals at the European Food Safety Authority: Principles, Guidance Documents, Applications and Future Challenges. Toxins.

[45] Rempelos L., Wang J., Baranski M., Watson A., Volakakis N., Hoppe H.W., Kuhn-Velten W.N., Hadall C., Hasanaliyeva G., Chatzidimitriou E. (2022). Diet and food type affect urinary pesticide residue excretion profiles in healthy individuals: Results of a randomized controlled dietary intervention trial. Am. J. Clin. Nutr..

[46] Vasylieva N., Barnych B., Wan D., El-Sheikh E.A., Nguyen H.M., Wulff H., McMahen R., Strynar M., Gee S.J., Hammock B.D. (2017). Hydroxy-fipronil is a new urinary biomarker of exposure to fipronil. Environ. Int..

[47] Andersen H.R., Rambaud L., Riou M., Buekers J., Remy S., Berman T., Govarts E. (2022). Exposure Levels of Pyrethroids, Chlorpyrifos and Glyphosate in EU-An Overview of Human Biomonitoring Studies Published since 2000. Toxics.

[48] EFSA (European Food Safety Authority) (2019). Statement on the available outcomes of the human health assessment in the context of the pesticides peer review of the active substance chlorpyrifos. EFSA J..

[49] Qiang S., Mohamed F., Mackenzie L., Roberts M.S. (2023). Rapid determination of polyethoxylated tallow amine surfactants in human plasma by LC-MSMS. Talanta.

[50] Mie A., Andersen H.R., Gunnarsson S., Kahl J., Kesse-Guyot E., Rembiałkowska E., Quaglio G., Grandjean P. (2017). Human health implications of organic food and organic agriculture: A comprehensive review. Environ. Health.

[51] Gómez-Ramos M.d.M., Nannou C., Martínez Bueno M.J., Goday A., Murcia-Morales M., Ferrer C., Fernández-Alba A.R. (2020). Pesticide residues evaluation of organic crops. A critical appraisal. Food Chem. X.

[52] Rebouillat P., Vidal R., Cravedi J.P., Taupier-Letage B., Debrauwer L., Gamet-Payrastre L., Touvier M., Hercberg S., Lairon D., Baudry J. (2021). Estimated dietary pesticide exposure from plant-based foods using NMF-derived profiles in a large sample of French adults. Eur. J. Nutr..

[53] Witczak A., Abdel-Gawad H. (2012). Comparison of organochlorine pesticides and polychlorinated biphenyls residues in vegetables, grain and soil from organic and conventional farming in Poland. J. Environ. Sci. Health. Part. B Pestic. Food Contam. Agric. Wastes.

[54] EC European Commission Regulation (EC) No 850/2004 of the European Parliament and of the Council of 29 April 2004 on Persistent Organic Pollutants and Amending Directive 79/117/EEC 2009. http://data.europa.eu/eli/reg/2004/850/2009-05-05.

[55] Sivaperumal P., Thasale R., Kumar D., Mehta T.G., Limbachiya R. (2022). Human health risk assessment of pesticide residues in vegetable and fruit samples in Gujarat State, India. Heliyon.

[56] Yao R., Yao S., Ai T., Huang J., Liu Y., Sun J. (2023). Organophosphate Pesticides and Pyrethroids in Farmland of the Pearl River Delta, China: Regional Residue, Distributions and Risks. Int. J. Environ. Res. Public Health.

[57] European Parliament and the Council Directive 2009/128/EC of the European Parliament and of the Council of 21 October 2009 Establishing a Framework for Community Action to Achieve the Sustainable Use of Pesticides. https://eur-lex.europa.eu/legal-content/EN/ALL/?uri=celex%3A32009L0128.

[58] Tataridas A., Kanatas P., Chatzigeorgiou A., Zannopoulos S., Travlos I. (2022). Sustainable Crop and Weed Management in the Era of the EU Green Deal: A Survival Guide. Agronomy.

[59] Helepciuc F.-E., Todor A. (2022). Greener European Agriculture? Evaluating EU Member States’ Transition Efforts to Integrated Pest Management through Their National Action Plans. Agronomy.

[60] Umetsu N., Shirai Y. (2020). Development of novel pesticides in the 21st century. J. Pestic. Sci..

[61] Damalas C.A., Koutroubas S.D. (2018). Current Status and Recent Developments in Biopesticide Use. Agriculture.

[62] Iammarino M., Panseri S., Unlu G., Marchesani G., Bevilacqua A. (2022). Editorial: Novel chemical, microbiological and physical approaches in food safety control. Front. Nutr..

[63] Iammarino M., Palermo C., Tomasevic I. (2022). Advanced Analysis Techniques of Food Contaminants and Risk Assessment-Editorial. Appl. Sci..

[64] EFSA-SANTE (2021). EFSA-SANTE Action Plan on Cumulative Risk Assessment for Pesticides Residues. Standing Committee for Plants, Animals, Food and Feed, Section Phytopharmaceuticals, Pesticide Residues. SANTE/10178/2021. https://ec.europa.eu/food/sites/food/files/plant/docs/pesticides_mrl_cum-risk-ass_sante-10178-2021.pdf.

[65] Rawtani D., Khatri N., Tyagi S., Pandey G. (2018). Nanotechnology-based recent approaches for sensing and remediation of pesticides. J. Environ. Manag..

[66] Zamora-Sequeira R., Starbird-Pérez R., Rojas-Carillo O., Vargas-Villalobos S. (2019). What are the Main Sensor Methods for Quantifying Pesticides in Agricultural Activities? A Review. Molecules.

[67] Ghosh S., AlKafaas S.S., Bornman C., Apollon W., Hussien A.M., Badawy A.E., Amer M.H., Kamel M.B., Mekawy E.A., Bedair H. (2022). The application of rapid test paper technology for pesticide detection in horticulture crops: A comprehensive review. Beni-Suef Univ. J. Basic Appl. Sci..

[68] Pelton R. (2009). Bioactive paper provides a low-cost platform for diagnostics. Trends Anal. Chem..

[69] Sicard C., Glen C., Aubie B., Wallace D., Jahanshahi-Anbuhi S., Pennings K., Daigger G.T., Pelton R., Brennan J.D., Filipe C.D.M. (2015). Tools for water quality monitoring and mapping using paper-based sensors and cell phones. Water Res..

[70] Phongphut A., Chayasombat B., Cass A.E.G., Phisalaphong M., Prichanont S., Thanachayanont C., Chodjarusawad T. (2022). Biosensors Based on Acetylcholinesterase Immobilized on Clay–Gold Nanocomposites for the Discrimination of Chlorpyrifos and Carbaryl. ACS Omega.

[71] Jia M., Zhombo E., Zhai F., Bing X. (2020). Rapid Multi-Residue Detection Methods for Pesticides and Veterinary Drugs. Molecules.

[72] Garvey J., Walsh T., Devaney E., King T., Kilduff R. (2020). Multi-residue analysis of pesticide residues and polychlorinated biphenyls in fruit and vegetables using orbital ion trap high-resolution accurate mass spectrometry. Anal. Bioanal. Chem..

[73] Hajrulai-Musliu Z., Uzunov R., Jovanov S., Jankuloski D., Stojkovski V., Pendovski L., Sasanya J.J. (2021). A new LC-MS/MS method for multiple residues/contaminants in bovine meat. BMC Chem..

[74] Pedersen M., Hakme E., Ninga E., Frandsen H.L. (2023). Analysis of veterinary drug- and pesticide residues in pig muscle by LC-QTOF-MS. Food Control..

[75] (2018). Foods of Plant Origin—Multimethod for the Determination of Pesticide Residues Using GC- and LC-Based Analysis Following Acetonitrile Extraction/Partitioning and Clean-Up by Dispersive SPE. Modular QuEChERS-Method.

[76] Ninga E., Lehotay S.J., Sapozhnikova Y., Lightfield A.R., Strahan G.D., Monteiro S.H. (2022). Analysis of pesticides, veterinary drugs, and environmental contaminants in goat and lamb by the QuEChERSER mega-method. Anal. Methods.

[77] Monteiro S.H., Lehotay S.J., Sapozhnikova Y., Ninga E., Moura Andrade G.C.R., Lightfield A.R. (2022). Validation of the QuEChERSER mega-method for the analysis of pesticides, veterinary drugs, and environmental contaminants in tilapia (Oreochromis Niloticus). Food Addit. Contam. Part A Chem. Anal. Control. Expo. Risk Assess.

[78] Sharma A., Kumar V., Shahzad B., Tanveer M., Sidhu G.P.S., Handa N., Kohli S.K., Yadav P., Bali A.S., Parihar R.D. (2019). Worldwide pesticide usage and its impacts on ecosystem. SN Appl. Sci..

[79] Nayak S.B., Sahoo A.K., Kolanthasamy E., Rao K., Kumar V., Singh J., Kumar P. (2020). Role of pesticide application in environmental degradation and its remediation strategies. Environmental Degradation: Causes and Remediation Strategies: Volume 1.

[80] Hussain S., Siddique T., Arshad M., Saleem M. (2009). Bioremediation and phytoremediation of pesticides: Recent advances. Crit. Rev. Environ. Sci. Technol..

[81] Ning J., Gang G., Bai Z., Hu Q., Qi H., Ma A., Zhuan X., Zhuang G. (2012). In situ enhanced bioremediation of dichlorvos by a phyllosphere *Flavobacterium* strain. Front. Environ. Sci. Eng..

[82] Ozdal M., Ozdal O.G., Algur O.F., Kurbanoglu E.B. (2017). Biodegradation of α-endosulfan via hydrolysis pathway by Stenotrophomonas maltophilia OG2. 3 Biotech.

[83] Ramu S., Seetharaman B. (2014). Biodegradation of acephate and methamidophos by a soil bacterium *Pseudomonas aeruginosa* strain Is-6. J. Environ. Sci. Health Part B.

[84] Huang Y., Xiao L., Li F., Xiao M., Lin D., Long X., Wu Z. (2018). Microbial Degradation of Pesticide Residues and an Emphasis on the Degradation of Cypermethrin and 3-phenoxy Benzoic Acid: A Review. Molecules.

[85] Wolicka D., Suszek A., Borkowski A., Bielecka A. (2009). Application of aerobic microorganisms in bioremediation in situ of soil contaminated by petroleum products. Bioresour. Technol..

[86] Tyagi M., da Fonseca M.M., de Carvalho C.C. (2011). Bioaugmentation and biostimulation strategies to improve the effectiveness of bioremediation processes. Biodegradation.

[87] Iyer R., Iken B., Damania A. (2013). A comparison of organophosphate degradation genes and bioremediation applications. Environ. Microbiol. Rep..

[88] Dvořák P., Nikel P.I., Damborský J., de Lorenzo V. (2017). Bioremediation 3.0: Engineering pollutant-removing bacteria in the times of systemic biology. Biotechnol. Adv..

[89] Briceño G., Palma G., Durán N. (2007). Influence of Organic Amendment on the Biodegradation and Movement of Pesticides. Crit. Rev. Environ. Sci. Technol..

[90] Choi M.K., Kim K.D., Ahn K.M., Shin D.H., Hwang J.H., Seong C.N., Ka J.O. (2009). Genetic and phenotypic diversity of parathion-degrading bacteria isolated from rice paddy soils. J. Microbiol. Biotechnol..

[91] Singh B.K., Walker A., Morgan J.A., Wright D.J. (2004). Biodegradation of chlorpyrifos by enterobacter strain B-14 and its use in bioremediation of contaminated soils. Appl. Environ. Microbiol..

[92] Chanika E., Georgiadou D., Soueref E., Karas P., Karanasios E., Tsiropoulos N.G., Tzortzakakis E.A., Karpouzas D.G. (2011). Isolation of soil bacteria able to hydrolyze both organophosphate and carbamate pesticides. Bioresour. Technol..

[93] Cycoń M., Wójcik M., Piotrowska-Seget Z. (2009). Biodegradation of the organophosphorus insecticide diazinon by *Serratia* sp. and *Pseudomonas* sp. and their use in bioremediation of contaminated soil. Chemosphere.

[94] Singh B.K., Walker A. (2006). Microbial degradation of organophosphorus compounds. FEMS Microbiol. Rev..

[95] Caceres T.P., Megharaj M., Naidu R. (2008). Biodegradation of the pesticide fenamiphos by ten different species of green algae and cyanobacteria. Curr. Microbiol..

[96] Abdelrazek M., Abozeid A., Eltholth M., Abouelenien F., El-Midany S., Moustafa N., Mohamed R. (2019). Bioremediation of a pesticide and selected heavy metals in wastewater from various sources using a consortium of microalgae and cyanobacteria. Slov. Vet. Res..

[97] Zhao R.-B., Bao H.-Y., Liu Y.-X. (2010). Isolation and Characterization of Penicillium oxalicum ZHJ6 for Biodegradation of Methamidophos. Agric. Sci. China.

[98] Zhao J., Zhao D., Han J. Isolation and Characterization of Dimethoate Degrading Phytopathogen Fungus from Soil. Proceedings of the 2009 3rd International Conference on Bioinformatics and Biomedical Engineering.

[99] Tian J., Dong Q., Yu C., Zhao R., Wang J., Chen L. (2016). Biodegradation of the Organophosphate Trichlorfon and Its Major Degradation Products by a Novel Aspergillus sydowii PA F-2. J. Agric. Food Chem..

[100] Chen S., Liu C., Peng C., Liu H., Hu M., Zhong G. (2012). Biodegradation of chlorpyrifos and its hydrolysis product 3,5,6-trichloro-2-pyridinol by a new fungal strain Cladosporium cladosporioides Hu-01. PLoS ONE.

[101] Jain R., Veena G., Singh K., Sheetal G. (2012). Isolation and characterization of monocrotophos degrading activity of soil fungal isolate Aspergillus Niger MCP1 (ITCC7782.10). Int. J. Environ. Sci..

[102] Gaber S.E., Hussain M.T., Jahin H.S. (2020). Bioremediation of diazinon pesticide from aqueous solution by fungal-strains isolated from wastewater. World J. Chem..

[103] Sethunathan N., Yoshida T. (1973). A *Flavobacterium* sp. that degrades diazinon and parathion. Can. J. Microbiol..

[104] Serdar C.M., Gibson D.T., Munnecke D.M., Lancaster J.H. (1982). Plasmid Involvement in Parathion Hydrolysis by *Pseudomonas diminuta*. Appl. Environ. Microbiol..

[105] Lyagin I., Efremenko E. (2021). Enzymes, Reacting with Organophosphorus Compounds as Detoxifiers: Diversity and Functions. Int. J. Mol. Sci..

[106] Theriot C.M., Grunden A.M. (2011). Hydrolysis of organophosphorus compounds by microbial enzymes. Appl. Microbiol. Biotechnol..

[107] Sogorb M.A., Vilanova E. (2002). Enzymes involved in the detoxification of organophosphorus, carbamate and pyrethroid insecticides through hydrolysis. Toxicol. Lett..

[108] Ragnarsdottir K.V. (2000). Environmental fate and toxicology of organophosphate pesticides. J. Geol. Soc..

[109] Haque M.A., Hong S.Y., Hwang C.E., Kim S.C., Cho K.M. (2018). Cloning of an organophosphorus hydrolase (opdD) gene of Lactobacillus sakei WCP904 isolated from chlorpyrifos-impregnated kimchi and hydrolysis activities of its gene product for organophosphorus pesticides. Appl. Biol. Chem..

[110] Jiang B., Zhang N., Xing Y., Lian L., Chen Y., Zhang D., Li G., Sun G., Song Y. (2019). Microbial degradation of organophosphorus pesticides: Novel degraders, kinetics, functional genes, and genotoxicity assessment. Environ. Sci. Pollut. Res..

[111] Alejo-Gonzalez K., Hanson-Viana E., Vazquez-Duhalt R. (2018). Enzymatic detoxification of organophosphorus pesticides and related toxicants. J. Pestic. Sci..

[112] Caldwell S.R., Raushel F.M. (1991). Detoxification of organophosphate pesticides using an immobilized phosphotriesterase from *Pseudomonas diminuta*. Biotechnol. Bioeng..

[113] Benning M.M., Shim H., Raushel F.M., Holden H.M. (2001). High resolution X-ray structures of different metal-substituted forms of phosphotriesterase from *Pseudomonas diminuta*. Biochemistry.

[114] Kang D.G., Li L., Ha J.H., Choi S.S., Cha H.J. (2008). Efficient cell surface display of organophosphorous hydrolase using N-terminal domain of ice nucleation protein in Escherichia coli. Korean J. Chem. Eng..

[115] Horne I., Sutherland T.D., Harcourt R.L., Russell R.J., Oakeshott J.G. (2002). Identification of an opd (organophosphate degradation) gene in an Agrobacterium isolate. Appl. Environ. Microbiol..

[116] Dawson R.M., Pantelidis S., Rose H.R., Kotsonis S.E. (2008). Degradation of nerve agents by an organophosphate-degrading agent (OpdA). J. Hazard. Mater..

[117] Anderson B., Phillips B., Hunt J., Largay B., Shihadeh R., Tjeerdema R. (2011). Pesticide and toxicity reduction using an integrated vegetated treatment system. Environ. Toxicol. Chem..

[118] Scott C., Begley C., Taylor M.J., Pandey G., Momiroski V., French N., Brearley C., Kotsonis S.E., Selleck M.J., Carino F.A. (2011). Free-Enzyme Bioremediation of Pesticides. Pesticide Mitigation Strategies for Surface Water Quality.

[119] DeFrank J.J., Cheng T.C. (1991). Purification and properties of an organophosphorus acid anhydrase from a halophilic bacterial isolate. J. Bacteriol..

[120] Jain M., Yadav P., Joshi B., Joshi A., Kodgire P. (2021). A novel biosensor for the detection of organophosphorus (OP)-based pesticides using organophosphorus acid anhydrolase (OPAA)-FL variant. Appl. Microbiol. Biotechnol..

[121] Singh B.K. (2009). Organophosphorus-degrading bacteria: Ecology and industrial applications. Nat. Rev. Microbiol..

[122] Kulshreshtha S., Mathur N., Bhatnagar P. (2014). Mushroom as a product and their role in mycoremediation. AMB Express.

[123] Pandey C., Prabha D., Negi Y.K., Prasad R. (2018). Mycoremediation of Common Agricultural Pesticides. Mycoremediation and Environmental Sustainability: Volume 2.

[124] Adenipekun C.O., Lawal R. (2012). Uses of mushrooms in bioremediation: A Review. Biotechnol. Mol. Biol. Rev..

[125] Hock W., Sisler H. (1969). Metabolism of Chloroneb by Rhizoctonia solani and Other Fungi. J. Agric. Food Chem..

[126] Singh H., Singh H. (2006). Fungal Degradation of Pesticides. Mycoremediation: Fungal Bioremediation.

[127] Katayama A., Matsumura F. (1993). Degradation of organochlorine pesticides, particularly endosulfan, by Trichoderma harzianum. Environ. Toxicol. Chem..

[128] George N., Chauhan P., Sondhi S., Saini S., Puri N., Gupta N. (2014). Biodegradation and Analytical Methods for Detection of Organophosphorous Pesticide: Chlorpyrifos. Int. J. Pure Appl. Sci. Technol..

[129] Rao A.V., Sethunathan N. (1974). Degradation of parathion by Penicillium waksmanii Zaleski isolated from flooded acid sulphate soil. Arch. Microbiol..

[130] Kim Y.-H., Ahn J.-Y., Moon S.-H., Lee J. (2005). Biodegradation and detoxification of organophosphate insecticide, malathion by *Fusarium oxysporum* f. sp. pisi cutinase. Chemosphere.

[131] Mir Z.A., Bharose R., Lone A.H., Malik Z.A. (2017). Review on phytoremediation: An ecofriendly and green technology for removal of heavy metals. Crop Res..

[132] Truua J., Truu J., Espenberg M., Nõlvak H., Juhanson J. (2015). Phytoremediation And Plant-Assisted Bioremediation In Soil And Treatment Wetlands: A Review. Open Biotechnol. J..

[133] Tonelli F.C.P., Tonelli F.M.P., Lemos M.S., Nunes N.A.d.M., Bhat R.A., Tonelli F.M.P., Dar G.H., Hakeem K. (2022). Chapter 3—Mechanisms of phytoremediation. Phytoremediation.

[134] Bhalla G., Bhalla B., Kumar V., Sharma A., Hadi Dehghani M., Karri R.R., Anastopoulos I. (2022). Chapter 16—Bioremediation and phytoremediation of pesticides residues from contaminated water: A novel approach. Pesticides Remediation Technologies from Water and Wastewater.

[135] Singh T., Singh D.K. (2017). Phytoremediation of organochlorine pesticides: Concept, method, and recent developments. Int. J. Phytoremediation.

[136] Velázquez-Fernández J.B., Martínez-Rizo A.B., Ramírez-Sandoval M., Domínguez-Ojeda D. (2012). Biodegradation and bioremediation of organic pesticides. Pestic.-Recent Trends Pestic. Residue Assay.

[137] Takkar S., Shandilya C., Agrahari R., Chaurasia A., Vishwakarma K., Mohapatra S., Varma A., Mishra A. (2022). Green technology: Phytoremediation for pesticide pollution. Phytoremediation Technology for the Removal of Heavy Metals and Other Contaminants from Soil and Water.

[138] Trapp S., Karlson U. (2001). Aspects of phytoremediation of organic pollutants. J. Soils Sediments.

[139] Misra N.N., Pankaj S.K., Walsh T., O’Regan F., Bourke P., Cullen P.J. (2014). In-package nonthermal plasma degradation of pesticides on fresh produce. J. Hazard. Mater..

[140] Zhang A.-A., Sutar P.P., Bian Q., Fang X.-M., Ni J.-B., Xiao H.-W. (2022). Pesticide residue elimination for fruits and vegetables: The mechanisms, applications, and future trends of thermal and non-thermal technologies. J. Future Foods.

[141] Hanafi A., Elsheshetawy H.E., Faied S.F. (2016). Reduction of pesticides residues on okra fruits by different processing treatments. J. Für Verbraucherschutz Und Leb..

[142] Ranjitha Gracy T.K., Sharanyakanth P.S., Radhakrishnan M. (2022). Non-thermal technologies: Solution for hazardous pesticides reduction in fruits and vegetables. Crit. Rev. Food Sci. Nutr..

[143] Yang L., Hai C., Zhang H., Feng C., Luo M., Zhou P., Leng J., Tian X., Zhao C., Lai B. (2023). Insights into the role of oxidation and adsorption for degradation of methyl parathion by ferrate (VI). J. Environ. Chem. Eng..

[144] Wang J., Yue W., Teng Y., Zhai Y., Zhu H. (2023). Degradation kinetics and transformation pathway of methyl parathion by δ-MnO2/oxalic acid reaction system. Chemosphere.

[145] Alhalili Z. (2023). Metal Oxides Nanoparticles: General Structural Description, Chemical, Physical, and Biological Synthesis Methods, Role in Pesticides and Heavy Metal Removal through Wastewater Treatment. Molecules.

[146] Kaushal J., Khatri M., Arya S.K. (2021). A treatise on Organophosphate pesticide pollution: Current strategies and advancements in their environmental degradation and elimination. Ecotoxicol. Environ. Saf..

[147] Xiao Q., Xuan X., Boczkaj G., Yoon J.Y., Sun X. (2022). Photolysis for the Removal and Transformation of Pesticide Residues During Food Processing: A State-of-the-Art Minireview. Front. Nutr..

[148] Abedi-Firoozjah R., Ghasempour Z., Khorram S., Khezerlou A., Ehsani A. (2021). Non-thermal techniques: A new approach to removing pesticide residues from fresh products and water. Toxin Rev..

[149] Velioglu Y., Fikirdeşici Ergen S., Aksu P., Altindağ A. (2018). Effects of Ozone Treatment on the Degradation and Toxicity of Several Pesticides in Different Grou. J. Agric. Sci..

[150] Alsager O.A., Alnajrani M.N., Alhazzaa O. (2018). Decomposition of antibiotics by gamma irradiation: Kinetics, antimicrobial activity, and real application in food matrices. Chem. Eng. J..

[151] Khedr T., Hammad A., Elmarsafy A., Halawa E., Soliman M. (2019). Degradation of some organophosphorus pesticides in aqueous solution by gamma irradiation. J. Hazard. Mater..

[152] Yang L., Zhou J., Feng Y. (2022). Removal of pesticide residues from fresh vegetables by the coupled free chlorine/ultrasound process. Ultrason. Sonochemistry.

[153] Zhou Q., Bian Y., Peng Q., Liu F., Wang W., Chen F. (2019). The effects and mechanism of using ultrasonic dishwasher to remove five pesticides from rape and grape. Food Chem..

[154] Pallares N., Sebastia A., Martinez-Lucas V., Gonzalez-Angulo M., Barba F.J., Berrada H., Ferrer E. (2021). High Pressure Processing Impact on Alternariol and Aflatoxins of Grape Juice and Fruit Juice-Milk Based Beverages. Molecules.

[155] Iizuka T., Maeda S., Shimizu A. (2013). Removal of pesticide residue in cherry tomato by hydrostatic pressure. J. Food Eng..

[156] Cherif M.M., Assadi I., Khezami L., Ben Hamadi N., Assadi A.A., Elfalleh W. (2023). Review on Recent Applications of Cold Plasma for Safe and Sustainable Food Production: Principles, Implementation, and Application Limits. Appl. Sci..

[157] Zhou R., Zhou R., Yu F., Xi D., Wang P., Li J., Wang X., Zhang X., Bazaka K., Ostrikov K. (2018). Removal of organophosphorus pesticide residues from Lycium barbarum by gas phase surface discharge plasma. Chem. Eng. J..

[158] Ranjitha Gracy T.K., Gupta V., Radhakrishnan M. (2019). Influence of low-pressure non-thermal dielectric barrier discharge (DBD) plasma on chlorpyrifos reduction in tomatoes. J. Food Process. Eng..

[159] Dorraki N., Mahdavi V., Ghomi H., Ghasempour A. (2016). Elimination of diazinon insecticide from cucumber surface by atmospheric pressure air-dielectric barrier discharge plasma. Biointerphases.

[160] Zheng Y., Wu S., Dang J., Wang S., Liu Z., Fang J., Han P., Zhang J. (2019). Reduction of phoxim pesticide residues from grapes by atmospheric pressure non-thermal air plasma activated water. J. Hazard. Mater..

[161] Arshad R.N., Abdul-Malek Z., Roobab U., Munir M.A., Naderipour A., Qureshi M.I., El-Din Bekhit A., Liu Z.-W., Aadil R.M. (2021). Pulsed electric field: A potential alternative towards a sustainable food processing. Trends Food Sci. Technol..

[162] Lozowicka B., Jankowska M., Hrynko I., Kaczynski P. (2016). Removal of 16 pesticide residues from strawberries by washing with tap and ozone water, ultrasonic cleaning and boiling. Environ. Monit. Assess..

[163] Akdemir Evrendilek G., Keskin E., Golge O. (2020). Interaction and multi-objective effects of multiple non-thermal treatments of sour cherry juice: Pesticide removal, microbial inactivation, and quality preservation. J. Sci. Food Agric..

[164] Tomer V. (2013). Vegetable Processing At Household Level: Effective Tool Against Pesticide Residue Exposure. IOSR J. Environ. Sci. Toxicol. Food Technol..

[165] Yuan Z., Yao J., Liu H., Han J., Trebse P. (2014). Photodegradation of organophosphorus pesticides in honey medium. Ecotoxicol. Environ. Saf..

[166] Choi S.W., Shahbaz H.M., Kim J.U., Kim D.-H., Yoon S., Jeong S.H., Park J., Lee D.-U. (2020). Photolysis and TiO2 Photocatalytic Treatment under UVC/VUV Irradiation for Simultaneous Degradation of Pesticides and Microorganisms. Appl. Sci..

[167] Yang L., Li M., Li W., Jiang Y., Qiang Z. (2018). Bench- and pilot-scale studies on the removal of pesticides from water by VUV/UV process. Chem. Eng. J..

[168] Savic J.Z., Petrovic S.Z., Leskovac A.R., Lazarevic Pasti T.D., Nastasijevic B.J., Tanovic B.B., Gasic S.M., Vasic V.M. (2019). UV-C light irradiation enhances toxic effects of chlorpyrifos and its formulations. Food Chem..

[169] Papagiannaki D., Medana C., Binetti R., Calza P., Roslev P. (2020). Effect of UV-A, UV-B and UV-C irradiation of glyphosate on photolysis and mitigation of aquatic toxicity. Sci. Rep..

[170] Baranda A.B., Fundazuri O., Martínez de Marañón I. (2014). Photodegradation of several triazidic and organophosphorus pesticides in water by pulsed light technology. J. Photochem. Photobiol. A Chem..

[171] El-Saeid M.H., Alotaibi M.O., Alshabanat M., Alharbi K., Altowyan A.S., Al-Anazy M. (2021). Photo-Catalytic Remediation of Pesticides in Wastewater Using UV/TiO_2_. Water.

[172] Kaur R., Singh D., Kumari A., Sharma G., Rajput S., Arora S., Kaur R. (2023). Pesticide residues degradation strategies in soil and water: A review. Int. J. Environ. Sci. Technol..

[173] Jafari S.J., Moussavi G., Hossaini H. (2016). Degradation and mineralization of diazinon pesticide in UVC and UVC/TiO_2_ process. Desalination Water Treat..

[174] Gupta V.K., Eren T., Atar N., Yola M.L., Parlak C., Karimi-Maleh H. (2015). CoFe_2_O_4_@TiO_2_ decorated reduced graphene oxide nanocomposite for photocatalytic degradation of chlorpyrifos. J. Mol. Liq..

[175] Li W., Zhao Y., Yan X., Duan J., Saint C.P., Beecham S. (2019). Transformation pathway and toxicity assessment of malathion in aqueous solution during UV photolysis and photocatalysis. Chemosphere.

[176] Di Vaio A., Boccia F., Landriani L., Palladino R. (2020). Artificial Intelligence in the Agri-Food System: Rethinking Sustainable Business Models in the COVID-19 Scenario. Sustainability.

[177] Tian Z., Wang J.W., Li J., Han B. (2021). Designing future crops: Challenges and strategies for sustainable agriculture. Plant J..

[178] European Union (2020). Farm to Fork Strategy. For a Fair, Healthy and Environmentally-Friendly Food System.

[179] McGinley J., Healy M.G., Ryan P.C., Harmon O’Driscoll J., Mellander P.E., Morrison L., Siggins A. (2023). Impact of historical legacy pesticides on achieving legislative goals in Europe. Sci. Total Environ..

[180] Kaur N., Khunger A., Wallen S.L., Kaushik A., Chaudhary G.R., Varma R.S. (2021). Advanced green analytical chemistry for environmental pesticide detection. Curr. Opin. Green Sustain. Chem..

[181] Chaudhary V., Rustagi S., Kaushik A. (2023). Bio-derived smart nanostructures for efficient biosensors. Curr. Opin. Green Sustain. Chem..

[182] Rani M., Yadav J., Chaudhary S., Shanker U. (2021). An updated review on synthetic approaches of green nanomaterials and their application for removal of water pollutants: Current challenges, assessment and future perspectives. J. Environ. Chem. Eng..

[183] Bala S., Garg D., Thirumalesh B.V., Sharma M., Sridhar K., Inbaraj B.S., Tripathi M. (2022). Recent Strategies for Bioremediation of Emerging Pollutants: A Review for a Green and Sustainable Environment. Toxics.

[184] Dangi A.K., Sharma B., Hill R.T., Shukla P. (2019). Bioremediation through microbes: Systems biology and metabolic engineering approach. Crit. Rev. Biotechnol..

[185] Dash D.M., Osborne W.J. (2023). A systematic review on the implementation of advanced and evolutionary biotechnological tools for efficient bioremediation of organophosphorus pesticides. Chemosphere.

[186] Patil A., Yesankar P., Bhanse P., Maitreya A., Kapley A., Qureshi A., Naeem M., Bremont J.F.J., Ansari A.A., Gill S.S. (2022). Omics Perspective: Molecular Blueprint for Agrochemical Bioremediation Process in the Environment. Agrochemicals in Soil and Environment: Impacts and Remediation.

[187] Hassan S., Ganai B.A. (2023). Deciphering the recent trends in pesticide bioremediation using genome editing and multi-omics approaches: A review. World J. Microbiol. Biotechnol..

[188] Sharma B., Shukla P. (2020). Designing synthetic microbial communities for effectual bioremediation: A review. Biocatal. Biotransformation.

[189] Zimny T. (2022). New genomic techniques and their European Union reform. Potential policy changes and their implications. Front. Bioeng. Biotechnol..

[190] Armenova N., Tsigoriyna L., Arsov A., Petrov K., Petrova P. (2023). Microbial Detoxification of Residual Pesticides in Fermented Foods: Current Status and Prospects. Foods.

[191] Petrova P., Arsov A., Tsvetanova F., Parvanova-Mancheva T., Vasileva E., Tsigoriyna L., Petrov K. (2022). The Complex Role of Lactic Acid Bacteria in Food Detoxification. Nutrients.

[192] Sachithra V., Subhashini L.D.C.S. (2023). How artificial intelligence uses to achieve the agriculture sustainability: Systematic review. Artif. Intell. Agric..

[193] Ghatrehsamani S., Jha G., Dutta W., Molaei F., Nazrul F., Fortin M., Bansal S., Debangshi U., Neupane J. (2023). Artificial Intelligence Tools and Techniques to Combat Herbicide Resistant Weeds&mdash;A Review. Sustainability.

[194] Azmi H.N., Hajjaj S.S.H., Gsangaya K.R., Sultan M.T.H., Mail M.F., Hua L.S. (2023). Design and fabrication of an agricultural robot for crop seeding. Mater. Today Proc..

[195] Yang J., Ma S., Li Y., Zhang Z. (2022). Efficient Data-Driven Crop Pest Identification Based on Edge Distance-Entropy for Sustainable Agriculture. Sustainability.

[196] Zhou Z., Majeed Y., Diverres Naranjo G., Gambacorta E.M.T. (2021). Assessment for crop water stress with infrared thermal imagery in precision agriculture: A review and future prospects for deep learning applications. Comput. Electron. Agric..

[197] Marvin H.J.P., Bouzembrak Y., Janssen E.M., van der Fels-Klerx H.J., van Asselt E.D., Kleter G.A. (2016). A holistic approach to food safety risks: Food fraud as an example. Food Res. Int..

